# Common Reactivity and Properties of Heme Peroxidases: A DFT Study of Their Origin

**DOI:** 10.3390/antiox12020303

**Published:** 2023-01-28

**Authors:** Daniel R. Ramos, Paul G. Furtmüller, Christian Obinger, Ángeles Peña-Gallego, Ignacio Pérez-Juste, J. Arturo Santaballa

**Affiliations:** 1 Chemical Reactivity & Photoreactivity Group (React!), Department of Chemistry, CICA & Faculty of Sciences, Universidade da Coruña, Campus da Zapateira, E-15071 A Coruña, Spain; 2 Departamento de Química Física, Universidade de Vigo, Campus Universitario Lagoas-Marcosende, E-36310 Vigo, Spain; 3 Institute of Biochemistry, Department of Chemistry, University of Natural Resources and Life Sciences, Muthgasse 18, A-1190 Vienna, Austria

**Keywords:** peroxidase, density functional calculations, compound I, compound II, ferryl oxygen, reduction potential

## Abstract

Electronic structure calculations using the density-functional theory (DFT) have been performed to analyse the effect of water molecules and protonation on the heme group of peroxidases in different redox (ferric, ferrous, compounds I and II) and spin states. Shared geometries, spectroscopic properties at the Soret region, and the thermodynamics of peroxidases are discussed. B3LYP and M06-2X density functionals with different basis sets were employed on a common molecular model of the active site (Fe-centred porphine and proximal imidazole). Computed Gibbs free energies indicate that the corresponding aquo complexes are not thermodynamically stable, supporting the five-coordinate Fe(III) centre in native ferric peroxidases, with a water molecule located at a non-bonding distance. Protonation of the ferryl oxygen of compound II is discussed in terms of thermodynamics, Fe–O bond distances, and redox properties. It is demonstrated that this protonation is necessary to account for the experimental data, and computed Gibbs free energies reveal p*K*_a_ values of compound II about 8.5–9.0. Computation indicates that the general oxidative properties of peroxidase intermediates, as well as their reactivity towards water and protons and Soret bands, are mainly controlled by the iron porphyrin and its proximal histidine ligand.

## 1. Introduction

The omnipresent heme-containing proteins and enzymes catalyse many different processes including electron transfer, oxygen binding, oxidation, oxygenation, and other reactions [1,2]. Heme *b* or post-translationally modified heme present in heme peroxidases serve to catalyse the hydrogen peroxide-mediated one- or two-electron oxidation of a variety of molecules including cations, anions, aromatic molecules, and even proteins. Over the course of evolution, four superfamilies of heme peroxidases emerged, differing in overall fold, enzymatic activity, and active site architecture (e.g., cysteine or histidine as proximal ligand) [3].

**Scheme 1:**
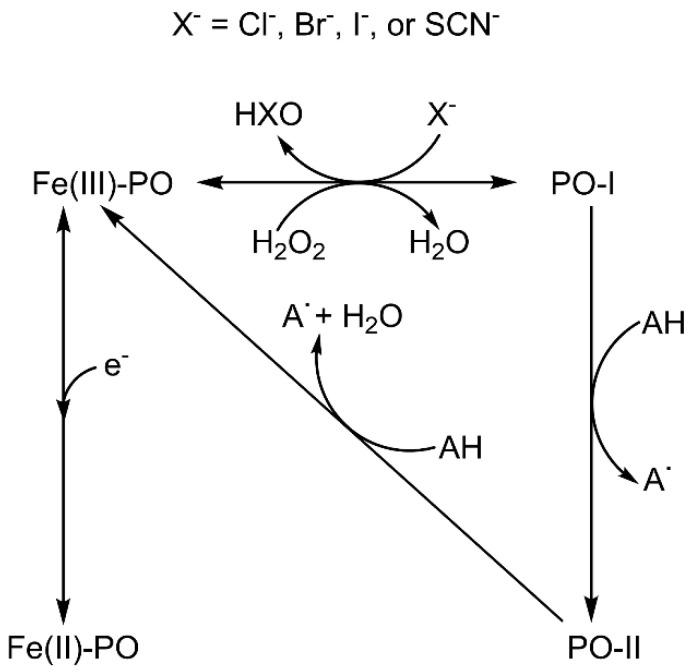
Peroxidase and chlorination cycles showing the interconversion between different species: Fe(III)-PO, ferric state; Fe(II)-PO, ferrous state; PO-I, compound I; PO-II, compound II. Note that the ferrous state does not participate in the reactions catalysed by heme peroxidases.

These enzymes work according to a multistep complex mechanism involving distinct redox intermediates (Scheme 1), which have been studied by several computational [4,5,6] and experimental [7,8] methods. Native ferric peroxidase (Fe(III)-PO) primarily uses hydrogen peroxide (H_2_O_2_), or other oxidants, to originate a ferryl (Fe(IV)=O) π-cation radical complex denominated compound I (PO-I), containing an oxygen atom generated from the heterolytic cleavage of H_2_O_2_ [9]. This intermediate can be one-electron reduced to compound II (PO-II), also containing a ferryl group. The ferrous state (Fe(II)-PO), which does not participate in a conventional peroxidase cycle nor play a role in their biological activity, can be produced by the reversible reduction of the ferric species.

Despite the fact that the four superfamilies show different structures in the active site, all heme peroxidases typically pass through the redox intermediates ferric state, compound I, and compound II during catalysis (Scheme 1). Differences are reported in the pH optimum of activity, the nature and binding site of the electron donor [3], the presence of distal water in the ferric state, as well as the protonation status of the ferryl oxygen in compound II [1,2,3,4,5,6,7,8,9].

Despite the fact that the optimum pH of many peroxidase activities is below 7, so far, less attention has been paid to the acidic range; in fact, many experimental studies on the reactivity and electronic structure of heme peroxidases have been performed under neutral or basic pH conditions. For instance, the oxidation of chloride by human myeloperoxidase (MPO) is much more efficient at pH 5 than at 7 [8,10,11,12,13]. Furthermore, the crystal structures of many peroxidases present a discrete water in the distal heme cavity in axial position to the iron atom [9]. Under acidic pH conditions, the presence of H_2_O and H^+^ in the distal cavity has to be considered, instead of that of HO^−^, which dominates at strong alkaline conditions.

In general, protons are directly involved in the reactivity of peroxidases (Scheme 1) and, as a consequence, ferryl centre protonation has been essential to justify the different Fe–O bond distances measured for compounds I and II by X-ray diffraction structural analysis [14]. Conversely, oxo-ferryl stretching frequencies determined by resonance Raman spectroscopy (RR) indicate that both species have similar short Fe–O distances [15,16], suggesting a non-protonated ferryl oxygen [17]. Studies on the extended X-ray absorption fine structure (EXAFS) also ratify non-protonated compound II [18]. However, the photoreduction of iron may take place upon X-ray irradiation and even by laser excitation, leading to some uncertainty about the actual iron oxidation state in these experiments [19,20,21]. In addition, some inconsistencies appear, for example the long Fe–O distance obtained by X-ray diffraction for cytochrome *c* peroxidase (CCP) compound I [22], or the short [18] and long [23] Fe–O distances determined by EXAFS for compound II of horseradish peroxidase (HRP). Protonated ferryl oxygen was observed for compound II of chloroperoxidase (CPO, a heme peroxidase with proximal thiolate) by RR [24] and EXAFS measurement [25], while compound II of myoglobin does not appear to be protonated even at pH 3.9 according to the same techniques [26]. The available neutron structures of the ferric and compound I intermediates of CCP [27] and compound II of ascorbate peroxidase (APX) [28] show the existence of a Fe(IV)-OH group only in the latter.

Peroxidases possess in common many properties and molecular structures at the active site. However, at least in three superfamilies, the only common part of all these proteins is the proximal imidazole coordinated to a Fe-centred porphine ring. The proximal histidine can occupy several positions and interactions with the protein matrix, which affect the rotation of imidazole as well as its charge distribution. Other examples of variability include the mode of interaction of heme substituents with the protein matrix or even the post-translational modification of those substituents as shown in representatives of the peroxidase-cyclooxygenase superfamily [2,3] that may cause heme distortion together with unique spectral and redox properties [29]. On the other hand, conserved amino acids are found on the distal side, such as the catalytic pairs histidine/arginine or aspartate/arginine, but their positions vary with respect to heme and also with respect to their interactions with the distal protein matrix [3]. Thus, it appears that the predominant behaviour of these enzymes actually derives from only a relatively small part of their active site, while the particular performance of a specific peroxidase arises from the rest of the active site and its surroundings. A deep mechanistic understanding of peroxidases requires, apart from studying the effect of the protein, the knowledge of the underlying chemistry of all relevant redox species, which cannot be achieved by experimental means alone. To unveil the detailed mechanism in (bio)chemistry [30], electronic structure calculations can be very useful as they can focus on just a small part of the system.

Already in the last decades of the last century, some attempts were made to computationally model the active centre of peroxidases. The pioneering works by Gilda H. Loew and collaborators, starting with very reduced molecular models, could explain the resting state of several heme proteins according to the geometry of their active centres [31] or the structure and formation of compound I [4,32]. In addition, many other studies on these enzymes have been performed afterwards: the protonation state of compound II has been examined by applying Badger’s rule to the same molecular model here used [17], and the electronic effect of covalent bonding on the particular features of myeloperoxidase has been investigated computationally [33], although the results were not fully convincing. More recently, several studies have gained insight into particular enzymes and redox intermediates with density-functional theory (DFT) methods, as a work on cytochrome *c* peroxidase using a similar molecular structure to the one employed here [34]; however, a comprehensive computational study on peroxidases and the origin of their shared properties has never been published.

The objective of this work is to correctly describe some common general features of peroxidases, such as protonation state and p*K*_a_ values, stability of aqueous complexes, and UV-vis spectra and reduction potentials, using electronic structure calculations at a computational level able to provide reliable data at low computational cost coupled with a suitable common molecular model. For this purpose, the electronic, geometrical, and thermodynamic parameters were calculated for a small model of the active site of the following redox intermediates of peroxidases: native ferric and ferrous species, compound I and compound II, and their aquo and protonated complexes. The computational results are compared with empirical data to verify the suitability of both the computational method and the molecular model, and to successfully elucidate the interactions with distal water molecules and protonation states. Furthermore, this research provides a simple method to achieve additional information about other different heme peroxidases that cannot be obtained by experimental techniques. It is also a suitable starting point for future computational studies of these enzymes from a general point of view, or for the analysis of some properties of specific enzymes by ad hoc variation of the molecular model used to consider their particular structure and reactivity.

## 2. Computational Details

### 2.1. Models

Different molecular models of the active site have been used in computational studies of heme proteins, ranging from a few atoms neighbouring the heme metal centre to others encompassing the entire protein [35]. Some studies have been carried out using the complete porphyrin plus the axial imidazole, as it was chosen here [17]. Peripheral substituents and their interaction with the protein may have an effect on the configuration of the heme; therefore, a common approach in electronic structure calculations on heme porphyrins is to use X-ray structures to account for such effects rather than including the interaction partners in demanding geometry optimisations [36]. However, doubts have arisen as to whether the crystal arrangements match those in solution; indeed, some differences exist between NMR measurements in solution and crystal geometries of cytochrome *c* [37].

In addition to the described general peroxidase behaviour, the enzymes of the four superfamilies [3] show different enzymatic activities under diverse optimal conditions. The origin of these differences has to do with subtle variations in the multistep reaction mechanisms, induced by the protein environment. The use of any specific empirical structure could lead to a specific property, not to the desired general behaviour of peroxidases, the aim of this study. On the other hand, geometry optimisation of isolated porphyrins may lead to electronic ground states different from those detected in proteins [38].

**Figure 1:**
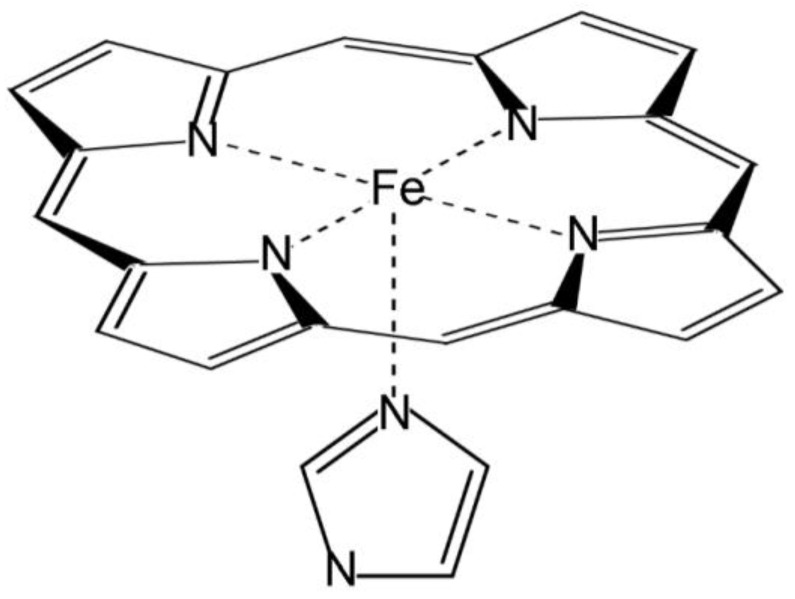
Molecular model of the active site of peroxidases.

In this work, the active site of heme peroxidases has been modelled employing Fe-centred porphine, i.e., a porphyrin ring without side chains, and an imidazole ring coordinated to the metal atom in axial position, mimicking the proximal histidine (Figure 1). The latter appears in three out of four heme peroxidase superfamilies [3]. This arrangement was chosen on the basis of the studies that indicate that the axial imidazole significantly increases the binding energy of ligands in comparison to the vinyl and propionate side chains [38,39,40], which, additionally, are modified in the peroxidase-cyclooxygenase superfamily [3]. In addition, the selected system is small enough for allowing the use of highly demanding basis sets in the calculations, and for accepting any necessary enlargements in further studies to examine other enzymatic complexes or some specific heme peroxidases. The distal sixth coordination site in ferric and ferrous intermediates is either vacant or eventually occupied by a water molecule, while in the case of the ferryl species compound I and compound II, an oxygen atom plays the role of the sixth ligand. According to crystallographic studies, water molecules (or related species) stay closer to the heme iron or ferryl oxygen than any surrounding amino acid residue. In addition, protonation processes may take place, and this computational study considers all possible scenarios (including untenable ferric and ferrous intermediates with protonated iron) for a comprehensive analysis. It should be noted that this small active site model ignores the effect of the protein on heme stabilisation, but it cancels out when comparing different species.

### 2.2. Methods

The adequacy of several DFT functionals for the correct calculation of the same model employed in this work, that is, an Fe-centred porphyrin ring coordinated to an imidazole group, has been assessed by comparing these methods with the results of computationally demanding high-level CCSD(T) calculations with large basis sets for several model iron compounds [41]. All the studied functionals properly match the optimised geometries with the experimental crystal structures. Moreover, the hybrid DFT functionals provided quite accurate relative energies of the different spin multiplicities at much lower computational cost. In addition, some results of the bond dissociation energies of iron ligands indicate that B3LYP describes more accurately than other functionals the bonding to ligands containing oxygen [41]. On the other hand, the M06-2X functional shows a better performance to calculate weak interactions [42], such as those found between heme and some other functional groups present in the distal cavity, such as water molecules. Spin and electron densities were also calculated for iron porphyrins using both DFT and coupled cluster methods [43,44,45]. The good agreement between the results underlines the soundness of DFT calculations to compare the relative energies and to evaluate the stability of the different states of the systems considered.

Therefore, DFT-based calculations were carried out with two hybrid functionals: the Becke’s three-parameter method [46] with the correlation functional of Lee, Yang, and Parr, incorporating non-local and local terms [47,48] (B3LYP), and the meta exchange-correlation variation of the M06 functional of Zhao and Truhlar that includes double non-local exchange, M06-2X [49]. Split-valence Pople basis sets [50] 6-31G(d,p), 6-311G(2d,2p) and 6-311++G(2d,2p), and correlation-consistent Dunning basis sets [51] cc-pVDZ, cc-pVTZ, Aug-cc-pVTZ and cc-pVQZ were employed for all atoms but for Fe, where an effective core potential [52] was used to substitute its core electrons with the LANL2DZ basis set.

All calculations were performed with the polarised continuum model (PCM) [53] to take into account the influence of water molecules present in the distal heme pocket. Water exerts a more substantial effect than the bulk protein on the four redox intermediates, since the solvent interacts more tightly with the oxo-ferryl group and the iron atom, and this interplay must vary significantly among the studied species. The cavity employed for the PCM calculations was created using atomic radii of the Universal Force Field (UFF). Gaussian default values were used for water as a solvent and all the parameters defining the spheres.

Geometry optimisation is necessary to properly reproduce the structures and, consequently, their thermodynamics. Thus, all considered species were geometrically optimised at B3LYP/cc-pVDZ. Zero-point energies and thermal corrections of enthalpies and Gibbs free energies at 298.15 K were calculated from the corresponding harmonic frequencies. Full geometry optimisation at low- and high-spin was performed for Fe(III)-PO, PO-I, and PO-II. The *d*^6^ Fe(II) atom can have three spin multiplicities: *S* = 0 (low-spin), *S* = 1 (intermediate-spin), and *S* = 2 (high-spin). Thus, here, data from these states were calculated for the ferrous species. Hereafter, the total spin will be referred to as *S* (Σ*s*) or the corresponding multiplicity. Additionally, some other relevant species were fully optimised and their frequencies calculated with both functionals and the seven selected basis sets.

The fifty lowest singlet excited states were used to obtain the excitation energies with the time-dependent DFT approach (TD-DFT). The convoluted UV-vis spectra were extracted from the Gaussian output files with the GaussView program [54], employing Gaussian distributions with a peak half-width of 2500 cm^−1^. This combination has been successfully applied to similar chemical systems [33] for the obtention of UV-vis spectra, which provided suitable Soret band peak heights for comparison with experimental values.

**Figure 2:**
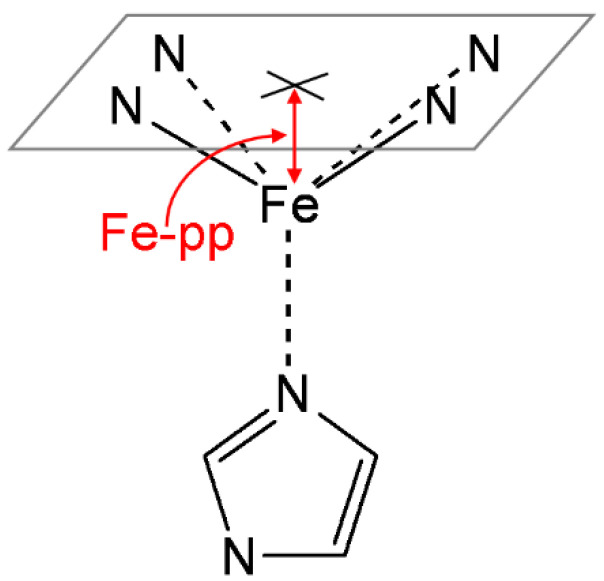
Iron out-of-plane distance (Fe–pp), defined as the distance between the plane determined by the four pyrrole nitrogen atoms and the iron.

The position of the iron atom with respect to the heme plane was calculated as shown in Figure 2. The best-fitting plane was determined from the four pyrrole nitrogen atoms (pyrrole plane), and the distance to Fe (Fe–pp) was then obtained. The relevant bond orders in all species considered were computed using the natural bond orbital (NBO) analysis of Weinhold et al. [55,56,57]. All calculations were carried out with the Gaussian 16 package [58].

### 2.3. Calculation of Thermodynamic Parameters

The dissociation equilibria of water complexes are defined by the following expression:

$$A-H_{2}O\left(\mathrm{aq},\;1\;M\right)\overset{\Delta {G^{{}^{\circ}}}_{A-H_{2}O}}{↔}A_{\left(\mathrm{aq},\;1\;M\right)}+H_{2}O_{\left(\mathrm{aq},\;1\;M\right)}$$

All the calculated values involving solvation have been corrected as for the standard state in aqueous solution. The concentration of water is commonly considered to be 55.5 M, but this is not applicable to the distal cavity of peroxidases, as only a few discrete water molecules were detected; therefore, one molar was also taken for water concentration.

Protonation processes thermodynamics is usually reported by the corresponding p*K*_a_ value. Its computational determination involves the optimised geometries and the corresponding Gibbs free energies of the protonated and non-protonated forms of the ionisable species under study, as indicated in the following acid-base equilibrium:

$$\mathrm{AH}\left(\mathrm{aq},\;1\;M\right)\overset{\Delta {G^{{}^{\circ}}}_{\mathrm{AH}}}{↔}A_{\left(\mathrm{aq},\;1\;M\right)}^{-}+H_{\left(\mathrm{aq},\;1\;M\right)}^{+}$$

The non-protonated form, designated A^−^, has a net charge one unit more negative than the protonated form, AH. The equation used to calculate the absolute p*K*_a_ is given by:

$$pK_{a}=\frac{\Delta {G^{{}^{\circ}}}_{\mathrm{AH}}}{2.303RT}$$

Again, the solvation values must be revised to consider the conversion of concentration units when moving from the gas phase to the aqueous phase (atm to M). For proton in the gas phase [59] and in aqueous solution [60,61] published thermodynamic parameters were used.

The absolute one-electron reduction potential of any compound A can be obtained from the corresponding reduction reaction:

$$A\left(\mathrm{aq},\;1\;M\right)+e_{\left(g\right)}\overset{\Delta {G^{{}^{\circ}}}_{A/A^{.-}}}{↔}A_{\left(\mathrm{aq},\;1\;M\right)}^{.-}$$

It must be considered that the gas phase is the standard state for e^−^; its Gibbs free energy was obtained from reference [62]. The absolute reduction potential is calculated as follows:

$$E^{{}^{\circ}}=-\frac{\Delta {G^{{}^{\circ}}}_{A/A^{.-}}}{nF}$$

where *F* is the Faraday constant and n stands for the number of electrons participating in the half-reaction. Reduction potentials are usually given in relation to the standard hydrogen electrode (SHE), which has an absolute reduction potential *E*° = 4.44 V at 298.15 K [63], although slightly different results are achieved when alternative reference values are used [64].

The computational determination of the p*K*_a_ values and the reduction potentials can be estimated with the direct methods mentioned above or with the isodesmic method, where results are improved by balancing the reactions with similar systems used as reference (at the same computational level) [65]. Therefore, phenol deprotonation (p*K*_a_ = 9.99) [66] was selected as the complementary reaction for the determination of all isodesmic p*K*_a_ values [67]. An advantage of this procedure is that the use of uncertain thermodynamic data for the proton is not required [68]. On the other hand, the average reduction potential of compound I to ferric species measured for some native peroxidases, without considering enzymes belonging to the peroxidase cyclooxygenase superfamily, (*E*° = 0.926 V) [69] was the reference employed to correct the potential [70] of the other reduction processes. Similarly, the corresponding value for the compound I to compound II reduction reaction (*E*° = 0.977 V) [69] was then used to calculate the compound I to ferric state reduction potential.

## 3. Results and Discussion

**Figure 3:**
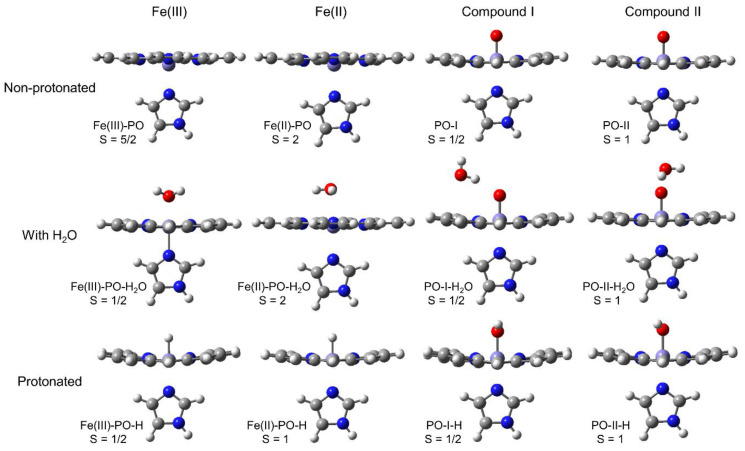
Structures optimised at the B3LYP/cc-pVDZ computational level for Fe(III) native peroxidase, ferrous species, compound I, and compound II at the most stable feasible spin state, either non-protonated, with a H_2_O discrete molecule, or protonated at a distal axial position.

All structures, fully optimised at the most stable spin state, are collected in Figure 3. The native ferric species (Fe(III)-PO) and the ferrous enzyme (Fe(II)-PO), as well as the aquo complex of the latter, are shown at high-spin state. The protonated ferrous species, Fe(II)-PO-H, corresponds to intermediate-spin. All other compounds present a low-spin state. The relevant bond orders, estimated from the corresponding Wiberg bond indices [71], and geometrical parameters are listed in Table S1 in Supplementary Materials. The corresponding representative published geometrical data for HRP [72,73], CCP [14,22,27], APX [14,28,74], LPO (lactoperoxidase) [75], and MPO [76] are also collected in Table S2, and used as experimental benchmarks throughout the paper for comparison. In this model, imidazole is not bound to the protein, therefore exhibiting free rotation around its axial position, while this motion would be much more restricted if proximal His and the entire peptide chain were taken into account. Previous computational studies have shown that this rotation only represents a few kJ mol^−1^ [77], so the orientation of this imidazole is not taken into account in the discussion. The relevant net Mulliken charges [78] determined at the B3LYP/cc-pVDZ level are shown in Table S3.

Relative Gibbs free energy values are listed in Table 1. The parent non-protonated species with the experimental most stable spin were employed as reference; their absolute free energies and other used values, are compiled in Table S4.

### 3.1. Non-Protonated Species

#### 3.1.1. Ferric and Ferrous Intermediates

According to B3LYP calculations, the high-spin Fe(III)-PO is 50 kJ mol^−1^ more stable than the doublet. In the case of Fe(II)-PO, a similar difference (46 kJ mol^−1^) between high- and low-spin states was found, whereas the *S* = 1 species lies 60 kJ mol^−1^ above the resting state. Non-protonated Fe(III)-PO and Fe(II)-PO share comparable geometries, even more similar if equivalent spin multiplicities are considered. A slightly out-of-plane Fe atom locates in proximal direction, more apparent in the high-spin states, while the planarity of the heme is preserved despite the metal shift. The iron to imidazole bond is clearly stronger for the low-spin states, as shown by the Fe–N_i_ (imidazole N) distances and bond orders. The distances between Fe and pyrrole nitrogens, Fe–N_p_ (pyrrole N), are almost the same for Fe(III)-PO and Fe(II)-PO, with minor deviations of just about 0.02 Å. The calculated values for both high-spin states fit well with the observed empirical ranges (except for the two representatives of the peroxidase-cyclooxygenase superfamily, namely LPO and MPO), but the outward position of the metal atom with respect to the heme plane is more evident in the computational data, probably due to the absence of distal interactions in the model. The obtained positive charge on Fe(III) atom is higher (ca. 0.3 a.u.) than on Fe(II). In addition, more negative values (approx. 0.6 a.u.) were determined for the porphine ring in Fe(II)-PO, which arise from the one-electron reduction in the transition from ferric to ferrous state, as well as the supposed gain of a proton in the heme moiety upon formation of this species.

#### 3.1.2. Compound I and Compound II

Calculations revealed low-spin species are significantly more stable (~50 kJ mol^−1^) for the hexacoordinate-Fe compounds PO-I and PO-II, which also exhibit very similar geometries. In these cases, Fe is hexacoordinate and forms a tight bond with ferryl oxygen (O_Fe_), characterised by short distances (≤1.63 Å) and bond orders higher than one (~1.4). This leads to a slightly longer Fe–N_i_ bond compared to Fe(III)-PO, especially in the case of PO-II. Similar Fe–O_Fe_ distances were determined for CCP compound I by both neutron diffraction and X-ray crystallography, while other enzymes show a somewhat longer distance. This bond arrangement implies an out-of-plane iron shift in the distal direction, which is strongly influenced by the spin state. For both compounds, the charge on O_Fe_ ranges between −0.30 and −0.35 a.u., which is largely compensated by a lower negative charge on the porphine ring and a slightly higher positive charge on the metal, compared to ferric and ferrous species. Compound I of most heme peroxidases has a porphyrin π-cation radical and, similarly, in the case of PO-I, there is a positive charge on this ring, which was only obtained in the protonated form of other oxidation states. In the real system, the charge on the porphyrin should be more positive, given the influence of the side chains of the heme and the interactions with the neighbouring adjacent protein. When ferryl oxygen is added to the model, the planarity of the heme is largely retained, although a slight saddle conformation is detected.

### 3.2. Aquo Complexes

#### 3.2.1. Ferric and Ferrous Intermediates

The addition of a single water molecule leads to minor molecular rearrangement, mainly a shift of Fe towards the porphyrin plane, which is more pronounced for the ferric species, resulting in some elongation of the Fe–N_i_ bond while the planarity of the heme is maintained. Therefore, the geometries obtained for ferric and ferrous aquo complexes are more similar than those of the bare species. Quite a weak bond is stablished between water oxygen (O_W_) and iron. The corresponding Wiberg bond indices are typical of hydrogen bonding. The Fe–O_W_ distance obtained for the ferric species is shorter than for most reference peroxidases, although a water molecule is even closer to the metal in APX. In contrast, calculations on the aquo-ferrous complex show the water molecule at a greater distance than in the experimental data. These complexes result from the photoreduction of compound I (containing a ferryl group), whereby ferryl oxygen is converted into water. The structures produced by direct reduction of the ferric species may be different, at least as far as the position of this water molecule is concerned. The water molecule is arranged axially in all optimised geometries, as evidenced by the ~180° angle to N_i_, like in the crystallographic structures; its net positive charge reveals charge transfer from the water molecule to the porphine ring.

The presence of water at the sixth coordination site stabilises preferentially the low-spin (and, to a minor extent, medium-spin) species, but the effect depends on the oxidation state. In the case of the ferric aquo complex, the low-spin state becomes much more stabilised due to the effective hexacoordination of Fe (Fe–O_W_ bond order = 0.39, almost twice the high-spin species, and in this case more charge is transferred) because the iron *d*-orbitals are better occupied for distal bonding. Thus, Fe(III)-PO with *S* = 5/2 is preferred, but the order reverses when considering a discrete water molecule. The water molecule bound to Fe(II)-PO exhibits an interaction with the metal atom that becomes tighter with decreasing spin values. This changes the relative stability of the spin states but the Gibbs free energy preference does not change. The addition of water also leads to a charge reallocation in the two species, the positive charge on the metal atom decreases as part of the electronic density of H_2_O is transferred; this effect is more obvious in Fe(III)-PO-H_2_O, where higher orders result for the Fe–O_W_ bond (Tables S1 and S3).

The obtained dissociation energies of aquo complexes, Δ*G*°_A-H_2_O_(aq), at the preferred spin multiplicities are summarised in Table 2. The negative value calculated for Fe(II)-PO-H_2_O indicates that this complex is unstable: bonding to ferrous centre is weak and Δ*G*°_A-H_2_O_(aq) = −5.12 kJ mol^−1^. In the case of Fe(III)-PO-H_2_O, the stabilization produced by the Fe–O interaction is weak but enough to compensate for the loss of aqueous PCM solvation energy, and the formation of the complex (at B3LYP/cc-pVDZ level) is favourable. However, the hydrogen bonds of the water molecule with other water molecules or with other available suitable residues in the distal cavity (typically 5 to 30 kJ mol^−1^ per H-bond) could preclude the formation of thermodynamically favourable water complexes. However, calculations with other basis sets (Table S5) yield negative Δ*G*°_A-H_2_O_(aq) values for this species, the more negative the bigger the basis employed. Furthermore, results obtained employing the M06-2X functional yield more positive numbers, which also decrease to very low values when increasing the basis set. Contrary to B3LYP results, structures calculated with M06-2X are more stable at high spin, and, therefore, Fe–O_W_ distances are longer and show weaker interactions. In summary, results with B3LYP plus (at least) triple-ζ basis sets and with M06-2X with any basis set, which are congruent with experimental values, show the pentacoordinate nature of the iron of ferric peroxidases, and the water molecule, although located in a distal axial position, at non-bonding distance.

#### 3.2.2. Compound I and Compound II

In contrast to the already commented heme-aquo complexes, the addition of a H-bonded water to PO-I and PO-II leads only to a slight polarisation of both the heme moiety and the water molecule. Some negative charge is transferred to the ferryl oxygen from the metalloporphyrin, while not significant electron-density transfer is observed from water, but this molecule also experiences some charge polarisation. The water molecule is not on the distal axis, the interacting H forms an angle to Fe of ~120°, typical of a *sp*^2^ hybridised orbital and associated to the electronic configuration of a double bonded oxygen atom (Fe=O) [79]. The Wiberg bond analysis gives a very low value for O_Fe_–H, indicating a weak H-bond, which is coupled with a slight change in the strength and length (i.e., a weakening) of the Fe–O_Fe_ ferryl bond and the opposite effect on the Fe–N_i_ bond. Nevertheless, computed forces appear stronger than in peroxidase crystals, where, moreover, the angles are different because the water molecules in the distal cavity are usually quite further away from O_Fe_ and become stabilised by other neighbouring interactions.

The dissociation energies are consequently affected, with Δ*G*°_A-H_2_O_(aq) values in the range −5 to −10 kJ mol^−1^, but the spin preference is not modified. The effects on the oxygen charge, and on the strength and length of the Fe–O_Fe_ double bond are quite similar for both ferryl intermediates. The obtained Fe–O_Fe_ distance is reasonable in the case of compound I (1.634 Å vs. experimental 1.635–1.743 Å), while the short elongation observed for this bond in compound II (also 1.634 Å) seems insufficient to reproduce the longer Fe–O_Fe_ distance obtained crystallographically for this species (1.830–1.879 Å). The interaction is weak and the dissociation process is favourable. However, in this case, the location of the water molecule (distance between iron and water oxygen > 3.8 Å) could allow a less strained cooperative interaction of this molecule with the ferryl moiety and other adequate residues situated above the heme in the distal cavity, becoming stabilised by extensive H-bonding. Thus, the water molecules located in the distal cavity can easily interact with compound I and compound II, while staying away from Fe in the ferric and ferrous intermediates, as this interaction is thermodynamically unstable. Furthermore, the differences between compound I and compound II cannot be explained by hydrogen bonds alone.

### 3.3. Protonated Species

Protonation exerts a more relevant effect on charge, geometry and spectral properties than that observed with water complexes. Thus, when a proton is added to the model, some negative charge is transferred from the heme moiety to H^+^, while only some bond polarization was observed with water and the electronic density transferred was much lower. This leads to some structural modification and subsequent thermodynamical changes.

#### 3.3.1. Ferric and Ferrous Intermediates

Protonation does not lead to a significant change in the heme structure. However, a shift in the iron position is produced, which becomes closer to the heme pyrrole plane. The bond to imidazole nitrogen weakens in low-spin species and the opposite effect is observed in high-spin structures. The formation of a bond between H^+^ and Fe in Fe(III)-PO-H and Fe(II)-PO-H (protonated ferric and ferrous intermediates, respectively) should be a very unfavourable process, since it requires a cation without any electron density available for donating to act as sixth ligand of Fe^3+^/Fe^2+^. However, the charge on this atom when bonded to Fe approaches zero for any spin state, but the high-spin ferric complex, while effective Fe–H bonding is observed (bond orders > 0.5). This electronic density has been fully transferred from the porphine ring. Protonation of ferrous and ferric intermediates leads to some heme-planarity distortion. The tighter the Fe–H bond, the more apparent this outcome, producing a heme saddle-shape conformation (the movement in the distal direction of two opposing meso carbon atoms of methine bridges while the other two shift to the proximal side), coupled with asymmetric Fe–N_p_ linkages.

Protonated ferric species shows a more stable low-spin state that arises from the hexacoordinate ferric iron, while the intermediate-spin state is more favoured for protonated ferrous intermediate. In addition, protonation involves a very significant destabilization effect on the ferric and ferrous species. Therefore, both Fe(III)-PO and Fe(II)-PO have very negative p*K*_a_ values, either calculated directly or with the isodesmic method (Table 2), which may be explained in terms of the high positive charge on Fe coupled with the partial desolvation of H^+^ in the distal cavity. Therefore, these two intermediates cannot be protonated on Fe even under extremely acidic aqueous conditions. Moreover, very low pH values drive to denaturalization and inactivation of the peroxidases, which would occur ahead of Fe protonation.

Accordingly, the experimentally observed protonation processes for ferric and ferrous intermediates should be attributed to other ionisable groups in or near the heme. In a computational study, Chiavarino et al. investigated the relative energies of a ferrous heme protonated at different positions [80]. The most stable compound (61.9 kJ mol^−1^) was obtained upon protonation at the β carbon atom of a vinyl group, and this site was then attributed to the proton acquired. In any case, the stabilisation observed relative to Fe(II)-PO-H would only account for an increase of ~10 p*K*_a_ units, still not enough to explain the proton addition in the formation of the ferrous species.

#### 3.3.2. Compound I and Compound II

Of great importance is the evident elongation obtained for Fe–O_Fe_, associated with a decrease in bond order, in the case of protonated compound I and compound II. The calculated distance for PO-I-H is longer than all experimental values, whereas the Fe–O_Fe_ bond distance obtained for PO-II-H (1.807 Å) agrees reasonably with the four structures of compound II (1.830–1.879 Å) used as reference. There is a concerted enhancement of the Fe–N_i_ interaction both in terms of distance and bond order when Fe undergoes a slight in-plane shift (PO-II-H at spin *S* = 2 reveals anomalous, but it also shows a weaker Fe–O_Fe_ bond than the other structures). The experimental data do not clearly show this effect. In addition, heme planarity becomes somewhat deformed, acquiring a saddle-shaped conformation, which is more evident at the lower spin multiplicities. The proton does indeed bind to O_Fe_ (bond order ~0.75) and charge transfer takes place from the heme, although in this case some proton character is retained, i.e., calculations give a residual positive charge of about 0.2 a.u. on this atom. Polarization is also detected with protonation. Thus, O_Fe_ slightly gains negative charge (<0.1 a.u) whereas the positive charge is mainly accumulated on the porphine ring. The elongation of the Fe–O_Fe_ bond leads to a different hybridisation of the O_Fe_ atom, becoming more like *sp*^3^, which is evident from the smaller Fe–O_Fe_–H angle. Nevertheless, a different behaviour was detected for the protonated compound II of APX (142°), possibly linked to other interactions within the distal cavity, although this proton does not point linearly to any other surrounding atom to form a H-bond, but the closeness of a water molecule may exert some steric effect.

The changes in charge distribution and geometry upon protonation of these two compounds are quite similar (except for the abnormal high-spin PO-II-H) and parallel the effects obtained with the addition of water, although to a greater extent. However, there is no change in the relative stability of the spin states, and the low-spin species are again favoured. The effective O_Fe_–H bond is the reason for the more stable free energies calculated for these protonated intermediates. It should be noted that the protonation stabilisation is always higher for compound II, and that the formation of PO-II-H is a spontaneous process (49 kJ mol^−1^).

The p*K*_a_ obtained for compound I with both methods is negative (Table 2), which means that this species is never protonated. Nevertheless, formation of PO-I-H is much more favourable than Fe(III)-PO-H and Fe(II)-PO-H. PO-II-H has a geometry very similar to that of PO-I-H, but has a lower Gibbs free energy compared to the non-protonated parent compound, leading to a positive p*K*_a_ value when calculated directly, but again negative when the calculation is performed with respect to phenol. Results obtained with other basis sets (Table S6) follow the same trend: negative values for PO-I-H, and around 8 for PO-II-H, which become mostly negative when calculated with the isodesmic method. However, this profile changes drastically when the M06-2X functional is employed. In this case, PO-I-H p*K*_a_ data range from 8 to 18 (direct method), decreasing to values close to 0 when calculated with respect to phenol deprotonation. These amended results also rule out the possibility of compound I protonation. On the other hand, p*K*_a_ values in the range 21–23 are obtained for PO-II-H, which reduce to 8–15 after the isodesmic correction. In both cases, this indicates that this compound is protonated at the normal enzyme function. These numbers may correspond to the range of p*K*_a_ values reported in the literature for compound II of HRP A (6.9) [15,81], other HRP isozymes (8.5) [15], or other peroxidases (~9) [82,83]. As previously mentioned, the protonation at the ferryl oxygen of compound II is still controversial. This process is often attributed to distal histidine, although the p*K*_a_ value corresponding to the imidazole deprotonation of free histidine is only 6.0, far below that obtained for peroxidases. Moreover, the neutral form of the imidazole inside a protein should be favoured. It does not seem plausible that the influence of a ferryl oxygen located 3.6–3.8 Å away from the N atom of the imidazole [14,72] could produce such a rise (2.5 to 3 p*K*_a_ units). On the other hand, the effect of this distal imidazole and the presence of some water molecules can lead to a more limited change in the p*K*_a_ of the ferryl oxygen (about ±1 unit). Even assuming that this effect is ignored in the molecular model employed and taking into account the error of the computational method, it can be considered that the p*K*_a_ value of about 8.5–9.0 observed for compound II could correspond to the ionisation of the protonated ferryl group. Recently, in another study, p*K*_a_ values were determined for a basic ferryl group in compound II of porphyrins [84], similar to the model employed in this work, but without the proximal imidazole. The influence of this coordination bond is of great importance, since hemoproteins with other proximal ligands have rather different activity and, apparently, the acidity of this proton also differs. Additionally, the character of the interactions of the ferryl group in the distal cavity must be different for PO-I and PO-II-H. We might suspect a different protonation state of the distal His, because the interaction with the non-protonated or protonated ferryl group should also exert different effects on the imidazole, although this is not consistent with the neutron diffraction results, where this moiety is protonated in both CCP compound I and APX compound II. However, it should be noted that there is not a porphyrin π-cation radical in the former but a proximal tryptophan radical [85].

Considering that compound I has a very low p*K*_a_ value and that the value determined for compound II could be equivalent to the experimentally obtained value of about 8.5–9.0 or even higher, the ferryl oxygen in these two compounds should be non-protonated (compound I) and protonated (compound II), as determined by neutron diffraction crystallography [27,28], at least for all the enzymes belonging to three heme peroxidases superfamilies.

### 3.4. Spectral Properties

Despite the specific structures of the different peroxidases, the Soret band of the four redox species is quite preserved in terms of position and intensity for the entire enzymatic family. The differences result from the particular protein environment of the heme group and, especially, from the covalent bonds between heme and protein. The Soret band of ferric peroxidases appears at ca. 405 nm with a molar absorption coefficient of about 100,000 cm^−1^·M^−1^. In compound I, the intensity at the same wavelength decreases to about 50%. In contrast, the Soret band of compound II presents an intensity similar to that of the ferric intermediate (~90,000 cm^−1^·M^−1^) but shifted to the 420 nm region. The ferrous state exhibits a more red-shifted Soret band (440 nm) with ε = 85,000 cm^−1^·M^−1^. All these values are averages of the experimental data found in the literature for some peroxidases after excluding exceptions, in particular MPO, which has three covalent linkages with the protein through two ester and one sulfonium bond, and a non-planar porphyrin group. As a result, MPO exhibits a particularly red-shifted band scheme [33,86].

**Figure 4:**
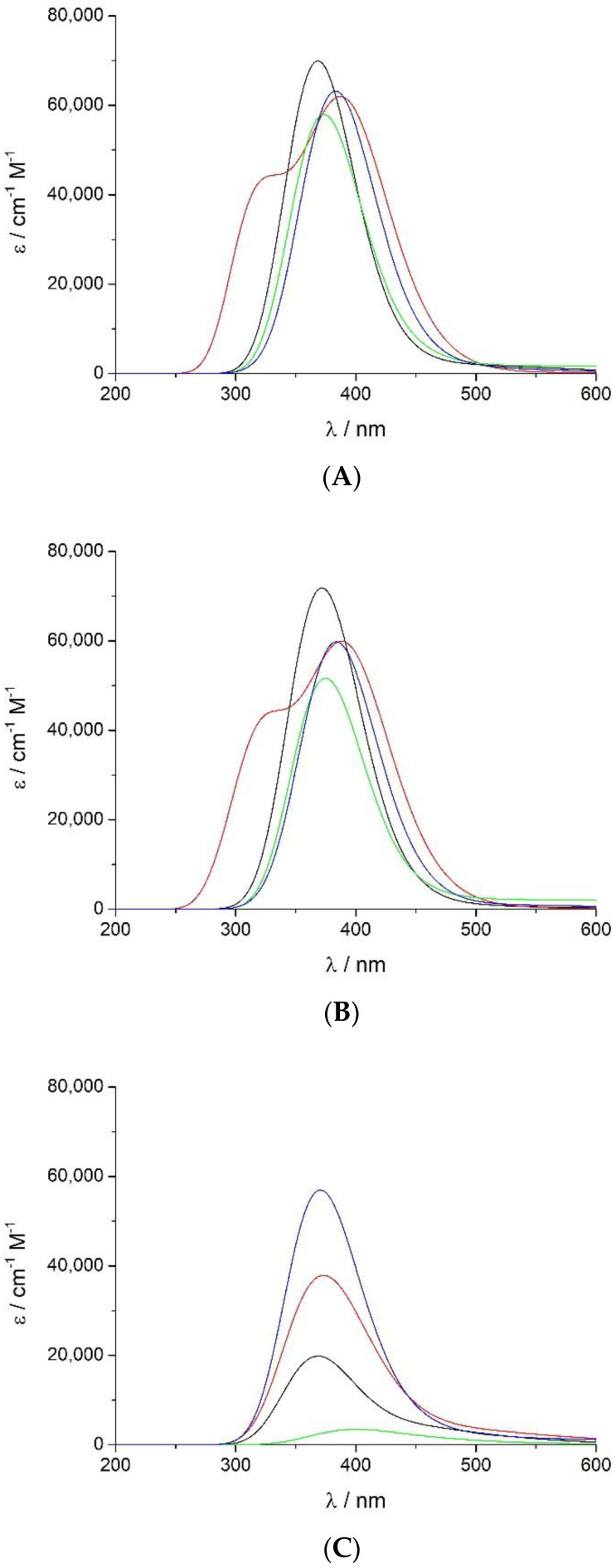
UV-vis spectra at the Soret region calculated by the TD-DFT functional with the B3LYP/cc-pVDZ computational level at the most stable spin state for (**A**) non-protonated Fe(III)-PO (─), Fe(II)-PO (─), PO-I (─), and PO-II (─); (**B**) corresponding aquo complexes; and (**C**) protonated species.

Figure 4A displays the calculated convoluted spectra in the Soret band region of the four non-protonated intermediates studied here at the most favourable spin state (further information is given in the Supplementary Materials, Table S7 and Figure S1). The absorption maxima show a net blue shift of 30–50 nm compared to the measured data, together with a suitable intensity profile. This blue shift has been described previously and arises from the proven poor performance of the TD-DFT functional used to describe the excitation energies [87,88], and from the lack of the porphyrin side chains in the molecular model [33]. Since the source of this divergence is the same, it is to be expected that the absolute error determined computationally is comparable for all compounds considered. Therefore, the relative wavelengths would more accurately reflect the experimental spectral shifts.

A minor red shift on the calculated Soret spectra was determined when the water molecule is included (Figure 4B), coupled with some reduction of the extinction coefficient, except for Fe(III)-PO-H_2_O, where it increases slightly. The predicted relative wavelength of PO-I is 5 nm higher than that of Fe(III)-PO (368 nm), but very similar to its aqueous complex (372 nm), and PO-I relative extinction coefficient is too high (58,000 cm^−1^ M^−1^), although lower than in both ferric species (70,000–72,000 cm^−1^ M^−1^). The spectrum of PO-I-H_2_O has less intensity (52,000 cm^−1^ M^−1^ at 375 nm) but still much higher than half the ferric peaks. Red shifts of 11–16 nm and 15–20 nm were obtained for compound II and ferrous complexes, respectively, with respect to ferric species. This behaviour is consistent with observed experimental red shifts, although to a lesser extent.

The calculated protonated species (Figure 4C) produce the Soret band spectra with very low intensity compared to the non-protonated enzymatic complexes and the corresponding experimental values. Only PO-II-H displays a relevant peak (57,000 cm^−1^ M^−1^ at 370 nm), which is not adequately red shifted; this could be related to the lack of some interactions with surrounding groups, missing in the computational model employed, and would need further analysis.

### 3.5. Redox Properties

Heme peroxidases are involved in one- and two-electron oxidation reactions. The native ferric species can be oxidised to compound I, which is either reduced directly by two-electron donors (for example, halides) to the ferric species or by one-electron donors to compound II and then to the ferric intermediate, which can also be reduced to ferrous state (Scheme 1). Both oxidised species have higher oxidation potential than the ferric and ferrous compounds. The oxidation-reduction properties of all these species have been determined for different peroxidases. The standard reduction potential of the Fe(III)/Fe(II) pair normally ranges from −120 to −310 mV [69]. Human myeloperoxidase is particular and displays a globin-like reduction potential of +5 mV [89] because of the presence of an electron withdrawing covalent sulfonium ion bond between a methionine and the heme group.

Compound I is the redox state with the highest oxidation potential. Values in the range of 750–950 mV and 900–1150 mV have been reported in heme *b* peroxidases for the reduction potential *E*°’ of the pairs compound I/ferric species and compound I/compound II, respectively [69]. However, more positive *E*°’ values of these couples have been obtained for the members of the peroxidase-cyclooxygenase superfamily, where the heme is post-translationally modified and covalently bound: 1150–1350 mV and 1000–1150 mV, respectively [90,91]. The reduction potentials for the compound II/ferric species pair range from 750 to 950 mV for heme *b* peroxidases and 950–1050 mV for the representatives of the peroxidase-cyclooxygenase superfamily [3]. All these values were measured under standard conditions (*T* = 298.15 K and pH = 7.0).

The reduction of native peroxidase and the redox steps included in the peroxidase cycle (the oxidation of ferric state to compound I, reduction of compound I to yield compound II and reduction of compound II to the ferric species) have been calculated theoretically:

Fe(III)-PO + e^−^ → Fe(II)-PO

Fe(III)-PO + H_2_O → PO-I + 2H^+^ + 2e^−^

PO-I + H^+^ + e^−^ → PO-II-H

PO-II-H + H^+^ + e^−^ → Fe(III)-PO + H_2_O

The corresponding standard reduction potentials relative to SHE, *E*°, have been computed for these redox processes (Table 3). Half reactions were modified to account for the protonated and non-protonated forms of compound II, while only the non-protonated species was considered for other intermediates.

Obtained reduction potential values calculated with the direct method are very dependent on the basis set and, particularly, on the functional employed (Table 3 and Table S8). Some of the obtained numbers differ considerably from the experimental ranges, but it can still be noticed that the overall redox profile resembles the experimental one only when protonated compound II is considered. It should be taken into account that, although some of these reduction potentials are pH dependent, all of them have been determined using the standard state of aqueous H^+^, i.e., 1 M. Therefore, the corresponding values at pH 7 would be slightly different, but the same findings would be derived from the data. On the other hand, isodesmic method results allow a better discussion. Reduction of PO-I to Fe(III)-PO yields *E*° values between 798 and 1069 mV with B3LYP, and somewhat lower with M06-2X (621 to 989). In both cases, reduction potentials increase with larger basis sets, so that, although all the values seem reasonable, the results obtained with M06-2X plus the best basis sets (triple-ζ at least) are very close to or within the experimental peroxidase range. However, in the case of the B3LYP functional, more adequate results are obtained with the lowest basis sets. Calculation of the reduction potential of the couple compound I/compound II yielded unsatisfactory results when the latter was non-protonated. On the other hand, the values calculated with PO-II-H range 834–1105 mV (B3LYP) and 914–1282 mV (M06-2X). In this case, the highest values were obtained with double-ζ basis sets and, again, the data that better mirror the experimental values were calculated with lesser and larger basis sets, for B3LYP and M06-2X, respectively. Similarly, the reduction potential of the redox pair compound II/ferric species calculated with non-protonated PO-II was too high (>1250 mV), and much more adequate data are obtained with PO-II-H, indicating again the protonation of compound II. As in the formation of compound I, the results increase with the basis set and they are closer to the experimental values with worse sets at B3LYP (747–918 mV) and with better ones at M06-2X (834–938 mV). It is necessary to mention here that, contrary to the direct method, the reduction potential values obtained with the isodesmic method and involving protonated compound II are pH independent. On the other hand, the reduction potentials involving non-protonated PO-II show some pH dependence, not enough to change the conclusions inferred. Finally, the values calculated for the reduction potential of the couple Fe(III)-PO/Fe(II)-PO with B3LYP (–373 to 237 mV) are more suitable than those of M06-2X (−698 to −1133 mV), which are substantially more negative than the empirical data. However, considering that the actual protonated ferrous intermediate, i.e., protonated on a different position than the iron, Fe(II)-PO-H*, must be more favourable than the non-protonated Fe(II)-PO, the calculated reduction potential would be less negative and closer to the experimental range. These values suggest that the origin of the oxidation-reduction properties of these peroxidase species mainly depends on the arrangement formed by the metalloporphyrin ring and the proximal imidazole.

### 3.6. Adequacy of the Computational Molecular Model and Methods

Our clear objective was to test the performance of a simple molecular model consisting of ferriporphine and an axial imidazole, the only part common to three of the four heme peroxidase superfamilies. The distal catalytic residues have not been included in this model because their formulation, position and distance to iron differ considerably in the enzymes from the four superfamilies. In addition, heme side chains are altered in the peroxidase-cyclooxygenase superfamily members, and the location and environment of proximal histidine differs significantly among peroxidases.

Very slight geometrical changes have been obtained among the different computational methods employed, but some substantial differences have been found in the thermodynamical parameters studied. The variations in the optimised geometries are insignificant to this work’s end and, more relevant, they do not affect the conclusions. The most important divergence in thermodynamical results occurs between the two functionals used. So, much more positive values have been obtained for Δ*G*°_A-H_2_O_(aq) of Fe(III)-PO-H_2_O and a more favoured high-spin aqueous complex with M06-2X. However, when combined with large basis sets, the results discard the possibility of a distal water molecule bonded to ferric iron, but agree with this axial water molecule situated at a non-bonding distance. The direct calculation of p*K*_a_s and standard reduction potentials yields defective values, which can be highly improved with the isodesmic method. To this end, the use of one of the half-reactions from the peroxidase cycle (two-electron reduction of PO-I to Fe(III)-PO) to correct the potential values of the other steps was quite successful. Consistent redox profiles were obtained with all computational levels, always provided that compound II is protonated; but the data calculated with M06-2X plus cc-pVTZ (or higher) fit better the experimental results. Furthermore, too low p*K*_a_ values were computed with B3LYP for PO-II-H, which do not support its protonation at physiological pH, and notably higher with M06-2X. Therefore, thermodynamical values calculated with the B3LYP functional are in general worse compared to those obtained with M06-2X, in particular when they are employed together with at least triple-ζ basis sets.

The results obtained for both the thermodynamics and the spectral and redox properties are adequate for most heme peroxidases, but they differ in the case of enzymes of the peroxidase-cyclooxygenase superfamily. These peroxidases contain hemes covalently linked to the protein: the prosthetic group is bonded to conserved Asp and Glu residues by two ester bonds [92]. As a result, the hemes are distorted and they exhibit some different chemical and biophysical features. Furthermore, MPO, which also has a sulfonium ion bond with Met243 [76], presents some additional distinctive properties: its heme groups are exceptionally bent, the Soret band is much red-shifted with respect to other peroxidases (which are usually red or brown coloured, while MPO is green), and it displays a particular redox profile that accounts for its proficiency in chloride oxidation to hypochlorous acid, a strong bactericidal agent. Thus, this enzyme is critical in host defense against infection [93].

All these particular properties are not supported by the current computational model. In fact, we do not pretend that the small molecular model of the active site employed here can replicate, accurately and in detail, the behaviour of any particular peroxidase, because it does not take into account the effects of heme-protein bonding, heme side chains, and overall protein environment. However, it contains the main shared structural attributes of their active sites and simulates their global behaviour and common features. In spite of their diverse protein structures, peroxidases have several physicochemical properties in common (comparable spectroscopic and oxidation-reduction features, active site geometry, reactivity, etc.), and the particular characteristics of a certain enzyme should be assigned to minor differences in the protein effect on the active site core. Thus, this plain molecular model in combination with a continuum solvation (PCM) at the B3LYP/cc-pVDZ computational level proved sufficient for the determination of a general geometry of the studied structures, while M06-2X/cc-pVTZ is necessary to reproduce satisfactorily several common thermodynamical properties of peroxidases. The latter method can be also successfully employed in computational studies with other similar systems.

## 4. Conclusions

Electronic structure calculations were performed to investigate the effects of hydrogen-bonded water molecules and protonation in the distal axial position of peroxidase intermediates. This is the first comprehensive computational study of ferric and ferrous species, compound I and compound II of peroxidases at several spin states, employing a simple molecular arrangement that is valid for all these enzymes. The use of this minimal molecular model coupled with B3LYP calculations to fully optimise the geometry and estimate the frequencies, with PCM water solvation to simulate the unspecific neighbouring interactions, and employing LANL2DZ for Fe and cc-PVDZ for other atoms, allowed a correct prediction of electronic ground states, as well as a reasonable description of the UV spectra at the Soret region. In addition, obtained geometries show a good correlation with experimental structures. To calculate adequate thermodynamic parameters, such as Δ*G*°_A-H_2_O_(aq) values, p*K*_a_ values, and reduction potentials (*E*°), the M06-2X/cc-pVTZ computational level is required.

Gibbs free energy values showed that the distal binding of a water molecule at the distal axial position of ferric species of peroxidases is thermodynamically unfavourable, which supports a five-coordinate Fe with this water molecule located axially but at a non-bonding distance to the ferric metal. In addition, unstable interactions with discrete water molecules were determined for the other redox states under study, as the aquo complexes are more energetic than the corresponding separate metalloporphyrin and water (Δ*G*°_A-H_2_O_(aq) < 0). Axial protonation on the distal side does not take place spontaneously under physiological conditions, as indicated by the low p*K*_a_ values obtained, with the exception of the more stable protonation of the ferryl oxygen in compound II. This proton accounts for the Fe–O_Fe_ elongation measured with X-ray diffraction experiments. In addition, this protonated compound is essential to explain the redox potentials for the reactions where it is involved. The present work proves that this must be a shared attribute of peroxidases and not just of APX, which was already established by neutron diffraction studies, and the estimated p*K*_a_ could correspond to the value of about 8.5–9.0 obtained experimentally for compound II, the first proposed value for this proton.

In conclusion, although the properties of specific enzymes depend on the surroundings and the heme-peptide bonds, the obtained results reveal the adequacy of both the molecular model used for characterising the most common features of peroxidases and the methodologies employed, which can be extended to other similar systems without available experimental data. The obtained agreement between experiments and calculations implies that the origin of the overall oxidation-reduction behaviour of heme peroxidases, along with their reactivity with protons and water molecules, as well as their Soret spectral properties, mainly lies in just the moiety comprised of iron porphine and the proximal histidine.

## Figures and Tables

**Table 1:** Gibbs free energy obtained with B3LYP/cc-pVDZ (*T* = 298.15 K) for all the studied species optimised at low, intermediate (for ferrous complexes only) and high spin. The most stable species of each non-protonated compound was used as benchmark. The relative values of protonated and aquo complexes were calculated with respect to the independent corresponding non-protonated species plus H^+^ or H_2_O, as applicable.

Species	Spin	Δ*G*°/kJ mol^−1^
Fe(III)-PO	1/2	49.73
	5/2	0.00
Fe(III)-PO-H_2_O	1/2	−12.44
	5/2	0.86
Fe(III)-PO-H	1/2	269.21
	5/2	307.07
Fe(II)-PO	0	46.38
	1	59.96
	2	0.00
Fe(II)-PO-H_2_O	0	25.66
	1	63.38
	2	5.13
Fe(II)-PO-H	0	231.01
	1	179.50
	2	221.61
PO-I	1/2	0.00
	5/2	49.61
PO-I-H_2_O	1/2	9.95
	5/2	59.44
PO-I-H	1/2	24.06
	5/2	86.53
PO-II	1	0.00
	2	56.93
PO-II-H_2_O	1	4.53
	2	62.60
PO-II-H	1	−48.66
	2	−9.19

**Table 2:** Gibbs free energy values calculated for the dissociation equilibrium of the studied aquo complexes (Δ*G*°_A-H_2_O_(aq)) in kJ mol^−1^, and p*K*_a_ values for the associated protonations, obtained directly (p*K*_a_^1^) and with the isodesmic method (p*K*_a_^2^). All data calculated by B3LYP/cc-pVDZ (*T* = 298.15 K). The most favourable spin states were employed; the first number under spin headers refers to non-protonated compound and the second value to the aquo complex or protonated species.

Species	Spin	Δ*G*°_A-H2O_(aq)	Spin	p*K*_a_^1^	p*K*_a_^2^
Fe(III)-PO	5/2–1/2	12.44	5/2–1/2	−47.2	−60.7
Fe(II)-PO	2–2	−5.12	2–1	−31.4	−45.0
PO-I	1/2–1/2	−9.95	1/2–1/2	−4.2	−17.7
PO-II	1–1	−4.53	1–1	8.5	−5.0

**Table 3:** Standard reduction potential (*E*°) values in mV relative to SHE calculated with direct and isodesmic methods, at B3LYP/cc-pVDZ and M06-2X/cc-pVTZ computational levels (*T* = 298.15 K). The most favourable spin states were employed (as specified in parentheses).

	Reduction Half-Reaction					
	Fe(III)-PO/Fe(II)-PO (5/2–2)	PO-I/Fe(III)-PO (1/2–5/2)	PO-I/PO-II (1/2–1)	PO-I/PO-II-H (1/2–1)	PO-II/Fe(III)-PO (1–5/2)	PO-II-H/Fe(III)-PO (1–5/2)
Direct method						
B3LYP/cc-pVDZ	377	1066	741	1245	1391	886
M06-2X/cc-pVTZ	662	2358	1120	2380	3596	2336
Isodesmic method						
B3LYP/cc-pVDZ	237	798	601	1105	1251	747
M06-2X/cc-pVTZ	−770	955	−312	948	2164	904

## Data Availability

Data are contained within the article or supplementary material.

## Supplementary Materials

The following supporting information can be downloaded at: https://www.mdpi.com/article/10.3390/antiox12020303/s1, Figure S1: Calculated excitation lines and the corresponding convoluted UV-vis absorption spectra at the Soret region obtained with the TD-DFT functional with the B3LYP/cc-pVDZ computational level at the most stable spin state for (A) non-protonated Fe(III)-PO, Fe(II)-PO, PO-I, and PO-II; (B) corresponding aquo complexes; and (C) protonated species; Table S1: Relevant geometrical parameters for all studied species fully optimized at the B3LYP/cc-pVDZ computational level for all considered spin states; Table S2: Relevant geometrical parameters obtained experimentally for selected peroxidases: horseradish peroxidase (HRP), cytochrome c peroxidase (CCP), ascorbate peroxidase (APX), lactoperoxidase (LPO), and myeloperoxidase (MPO); Table S3: Mulliken atomic charges (a.u.) on relevant atoms obtained at the B3LYP/cc-pVDZ computational level; Table S4: Gibbs free energy obtained with B3LYP/cc-pVDZ (*T* = 298.15 K) for unprotonated species at ground spin state, and electron and proton published values employed in the study. Absolute values used as reference for data in Table 1; Table S5: Gibbs free energy values in kJ mol^−1^ for the dissociation equilibrium of Fe(III)-PO-H_2_O (Δ*G*°_A-H_2_O_(aq)), and distance values (Å) between iron and water oxygen (Fe–O_W_) obtained for the aqueous complex, corresponding bond orders are shown. Data calculated by B3LYP and M06-2X functionals, with different basis sets; Table S6: p*K*_a_ values obtained for PO-I-H and PO-II-H through direct calculation (p*K*_a_^1^) and with the isodesmic method (p*K*_a_^2^). Data calculated by B3LYP and M06-2X functionals, with different basis sets; Table S7: Wavelength (nm) and molar absorption coefficient (cm^−1^·mol^−1^·dm^3^) of the Soret band obtained for all considered species and calculated with the B3LYP/cc-pVDZ computational method; Table S8: Standard reduction potential (*E*°) values in mV relative to SHE calculated with direct and isodesmic methods, by B3LYP and M06-2X functionals, with different basis sets.

## Abbreviations

HRP: horseradish peroxidase; MPO, myeloperoxidase; CCP, cytochrome *c* peroxidase; CPO, chloroperoxidase; APX, ascorbate peroxidase; LPO, lactoperoxidase; SHE, standard hydrogen electrode; Fe(III)-PO, native ferric peroxidase; Fe(II)-PO, ferrous peroxidase; PO-I, peroxidase compound I; PO-II, peroxidase compound II; Fe(III)-PO-H_2_O, Fe(II)-PO-H_2_O, PO-I-H_2_O, and PO-II-H_2_O, aquo complexes of previous species, respectively; Fe(III)-PO-H, Fe(II)-PO-H, PO-I-H, and PO-II-H, protonated forms, respectively; Fe(II)-PO-H*, ferrous peroxidase protonated on a different position than the iron.
