# Supplementary material for: Common Reactivity and Properties of Heme Peroxidases: A DFT Study of Their Origin
Source: Antioxidants (Basel). 2023 Jan 28;12(2):303. doi: 10.3390/antiox12020303 (PMC9952403; doi:10.3390/antiox12020303)
Supplement: Supplementary file 1 [file antioxidants-12-00303-s001.zip › antioxidants-2053590-supplementary.pdf]

Supplementary Information

Common reactivity and properties of heme peroxidases: a DFT study of their origin

Daniel R. Ramos *, Paul G. Furtmüller, Christian Obinger, Ángeles Peña-Gallego, Ignacio Pérez-Juste, J. Arturo Santaballa *

Table S1. Relevant geometrical parameters for all studied species fully optimized at the B3LYP/cc-pVDZ computational level for all considered spin states. Distance (Å) between iron and imidazole nitrogen atom (Fe–N_i), the metal centre and either ferryl or water oxygen (Fe–O) or proton (Fe–H), and between ferryl oxygen and proton or hydrogen atom from the water molecule (O–H); mean distance (Å) between iron and the four pyrrole nitrogen atoms (Fe–N_p); and distance (Å) from Fe atom to heme pyrrole plane (Fe–pp). Positive values of the latter indicate displacement towards distal side, while negative figures stand for out-of-plane Fe placed at the opposite side. Bond orders are shown in parentheses, values under Fe–N_p account for total iron-pyrrole nitrogen bond order, *i.e.* the overall order of those four covalent bonds. N_i–Fe–O, N_i–Fe–H, and Fe–O–H stand for the angles (°) among these atoms.

**Species**	**Spin**	**Fe–N_i**	**Fe–N_p**	**Fe–pp**	**Fe–O/H**	**O–H**	**N_i–Fe–O/H**	**Fe–O–H**
Fe(III)-PO	1/2	1.924 (0.63)	1.997 (2.24)	–0.151				
	5/2	2.093 (0.33)	2.075 (1.52)	–0.391				
Fe(III)-PO-H_2O	1/2	1.948 (0.60)	2.012 (2.22)	–0.063	2.056 (0.39)		179.54	
	5/2	2.156 (0.32)	2.064 (1.47)	–0.139	2.304 (0.22)		179.25	
Fe(III)-PO-H	1/2	2.111 (0.39)	2.002 (2.05)	–0.100	1.493 (0.74)		179.67	
	5/2	2.093 (0.37)	2.070 (1.53)	–0.324	1.545 (0.50)		177.25	
Fe(II)-PO	0	1.974 (0.55)	2.022 (2.01)	–0.150				
	1	2.387 (0.21)	2.020 (1.90)	–0.103				
	2	2.213 (0.26)	2.103 (1.24)	–0.327				
Fe(II)-PO-H_2O	0	2.024 (0.49)	2.029 (2.00)	–0.059	2.123 (0.35)		179.28	
	1	2.395 (0.21)	2.021 (1.90)	–0.088	2.861 (0.11)		170.99	
	2	2.220 (0.26)	2.101 (1.23)	–0.295	2.907 (0.09)		169.51	
Fe(II)-PO-H	0	2.149 (0.37)	2.013 (2.07)	–0.048	1.483 (0.79)		179.79	
	1	2.136 (0.37)	2.011 (2.07)	–0.079	1.493 (0.76)		179.73	
	2	2.135 (0.35)	2.084 (1.36)	–0.169	1.507 (0.70)		179.93	
PO-I	1/2	2.149 (0.35)	2.023 (2.04)	0.091	1.630 (1.42)		179.80	
	5/2	2.152 (0.34)	2.085 (1.38)	0.126	1.627 (1.42)		179.77	
PO-I-H_2O	1/2	2.141 (0.36)	2.021 (2.06)	0.094	1.634 (1.39)	1.845 (0.04)	179.82	126.15
	5/2	2.144 (0.35)	2.083 (1.40)	0.127	1.631 (1.37)	1.875 (0.04)	179.81	124.56
PO-I-H	1/2	2.056 (0.45)	2.017 (2.00)	0.021	1.800 (0.91)	0.973 (0.76)	176.95	110.93
	5/2	2.160 (0.35)	2.059 (1.56)	0.089	1.810 (0.82)	0.973 (0.74)	178.52	112.79
PO-II	1	2.193 (0.32)	2.024 (2.09)	0.111	1.628 (1.44)		179.84	
	2	2.207 (0.30)	2.085 (1.43)	0.163	1.629 (1.42)		179.86	
PO-II-H_2O	1	2.178 (0.33)	2.022 (2.10)	0.112	1.634 (1.40)	1.798 (0.05)	179.62	121.73
	2	2.196 (0.31)	2.083 (1.44)	0.163	1.635 (1.37)	1.827 (0.05)	179.52	121.32
PO-II-H	1	2.069 (0.43)	2.025 (2.03)	0.030	1.807 (0.90)	0.972 (0.77)	178.06	109.19
	2	2.389 (0.22)	2.034 (1.86)	0.138	1.903 (0.63)	0.971 (0.77)	179.76	113.72

Table S2. Relevant geometrical parameters obtained experimentally for selected peroxidases: horseradish peroxidase (HRP), cytochrome c peroxidase (CCP), ascorbate peroxidase (APX), lactoperoxidase (LPO), and myeloperoxidase (MPO). Distance (Å) between iron and imidazole nitrogen atom (Fe–N_i), the metal centre and either ferryl or water oxygen (Fe–O), and between ferryl oxygen and its proton (O–H); mean distance (Å) between iron and the four pyrrole nitrogen atoms (Fe–N_p); and distance (Å) from Fe atom to heme pyrrole plane (Fe–pp). Positive values of the latter indicate displacement towards distal side, while negative figures stand for out-of-plane Fe placed at the opposite side. N_i–Fe–O and Fe–O–H stand for the angles (°) among these atoms.

Species	Peroxidase	PDB	Fe–N_i	Fe–N_p	Fe–pp	Fe–O	O–H	N_i–Fe–O	Fe–O–H
Ferric	HRP	1W4W[a]	2.093	2.021	–0.140	–		–	
	CCP	1ZBY[b]	2.107	2.039	–0.274	2.330		176.15	
	CCP*	4CVI[c]	2.024	2.055	–0.181	2.651		169.64	
	APX	1OAG[d]	2.018	2.038	–0.199	1.944		176.90	
	LPO	2GJ1[e]	2.145	2.051	–0.203	–		–	
	MPO	1CXP[f]	2.187	1.989	–0.321	2.899		178.06	
			2.189	1.986	–0.342	3.000		172.99	
Ferrous	HRP	1H58[g]	2.133	2.028	–0.245	–		–	
	CCP	2XJ8[h]	2.062	2.117	–0.253	2.017		175.54	
	APX	2XJ6[h]	2.013	2.104	–0.220	2.306		177.77	
Compound I	HRP	1HCH[g]	2.141	2.009	0.028	1.705		177.05	
	CCP	2XIL[h]	2.099	2.050	0.077	1.635		178.92	
	CCP*	4CVJ[c]	2.124	2.039	0.098	1.645		169.51	
	APX	2XI6[h]	2.033	2.092	–0.087	1.743		176.11	
Compound II	HRP	1H55[g]	2.140	2.007	0.003	1.839		176.41	
	CCP	2XJ5[h]	2.121	2.070	–0.061	1.834		178.65	
	APX	2XIF[h]	1.994	2.027	–0.134	1.830		178.12	
	APX*	5JPR[i]	1.970	2.006	–0.081	1.879	0.986	178.76	142.28

* Structures obtained by neutron diffraction. [a] Ref. 73. [b] Ref. 22. [c] Ref. 27. [d] Ref. 74. [e] Ref. 75. [f] Ref. 76. [g] Ref. 72. [h] Ref. 14. [i] Ref. 28.

Table S3. Mulliken atomic charges (a.u.) on relevant atoms obtained at the B3LYP/cc-pVDZ computational level. N_i, N_p, O_{Fe}, and Por refer to imidazole nitrogen, total charge on the four pyrrole nitrogens, ferryl oxygen, and porphine, respectively.

Species	Spin	Fe	N_i	N_p	O_{Fe}	H	Por	H_2O
Fe(III)-PO	1/2	0.64	−0.26	−1.63			−0.06	
	5/2	0.89	−0.35	−1.87			−0.23	
Fe(III)-PO-H_2O	1/2	0.32	−0.23	−1.43			−0.11	0.33
	5/2	0.66	−0.28	−1.76			−0.25	0.24
Fe(III)-PO-H	1/2	0.21	−0.26	−1.35		0.11	1.34	
	5/2	0.54	−0.35	−1.74		0.32	0.79	
Fe(II)-PO	0	0.34	−0.23	−1.39			−0.63	
	1	0.43	−0.21	−1.57			−0.62	
	2	0.59	−0.28	−1.68			−0.82	
Fe(II)-PO-H_2O	0	0.11	−0.19	−1.26			−0.66	0.25
	1	0.26	−0.20	−1.54			−0.59	0.13
	2	0.41	−0.26	−1.66			−0.77	0.10
Fe(II)-PO-H	0	0.18	−0.22	−1.38		0.05	0.46	
	1	0.32	−0.27	−1.50		0.13	0.24	
	2	0.17	−0.25	−1.34		0.07	0.45	
PO-I	1/2	0.29	−0.19	−1.28	−0.30		0.63	
	5/2	0.46	−0.20	−1.44	−0.30		0.44	
PO-I-H_2O	1/2	0.29	−0.18	−1.28	−0.35		0.66	0.00
	5/2	0.46	−0.19	−1.43	−0.34		0.47	0.00
PO-I-H	1/2	0.26	−0.21	−1.23	−0.32	0.19	1.44	
	5/2	0.40	−0.22	−1.35	−0.36	0.20	1.36	
PO-II	1	0.26	−0.18	−1.34	−0.34		−0.25	
	2	0.43	−0.18	−1.51	−0.35		−0.43	
PO-II-H_2O	1	0.26	−0.17	−1.33	−0.38		−0.20	−0.02
	2	0.42	−0.18	−1.49	−0.39		−0.38	−0.01
PO-II-H	1	0.22	−0.20	−1.25	−0.34	0.18	0.54	
	2	0.41	−0.22	−1.40	−0.43	0.17	0.58	

Table S4. Gibbs free energy obtained with B3LYP/cc-pVDZ (T = 298.15 K) for unprotonated species at ground spin state, and electron and proton published values employed in the study. Absolute values used as reference for data in Table 1.

Species	Spin	$\Delta G°$ / kJ mol^{-1}
e^-	1/2	−3.632[a]
H^+	1/2[b]	−1130.90[c]
Fe(III)-PO	5/2	−3512299.76
Fe(II)-PO	2	−3512768.13
PO-I	1/2	−3709611.90
PO-II	1	−3710115.40
H_2O	0	−200643.65

[a] Ref. 62. [b] Nuclear spin. [c] Refs. 59-61.

Table S5. Gibbs free energy values in kJ mol^{-1} for the dissociation equilibrium of Fe(III)-PO-H_2O ($\Delta G°_{A\text{-}H_2O}$(aq)), and distance values (Å) between iron and water oxygen (Fe–O_W) obtained for the aqueous complex, corresponding bond orders are shown in parentheses. Data calculated by B3LYP and M06-2X functionals, with different basis sets for all atoms but Fe (LANL2DZ), and PCM at T = 298.15 K. The most favourable spin multiplicities were used; Fe(III)-PO with S = 1/2, and Fe(III)-PO-H_2O with S = 1/2 (B3LYP) or 5/2 (M06-2X).

**B3LYP**	**$\Delta G°_{A\text{-}H_2O}$(aq)**	**Fe–O_W**
6-31G(d,p)	–0.34	2.054 (0.38)
6-311G(2d,2p)	–7.29	2.068 (0.37)
6-311++G(2d,2p)	–31.29	2.070 (0.36)
cc-pVDZ	12.44	2.056 (0.39)
cc-pVTZ	–16.20	2.066 (0.36)
Aug-cc-pVTZ	–36.15	2.043 (0.37)
cc-pVQZ	–26.38	2.054 (0.37)
**M06-2X**	**$\Delta G°_{A\text{-}H_2O}$(aq)**	**Fe–O_W**
6-31G(d,p)	23.18	2.186 (0.23)
6-311G(2d,2p)	19.81	2.196 (0.22)
6-311++G(2d,2p)	2.35	2.201 (0.21)
cc-pVDZ	32.91	2.189 (0.24)
cc-pVTZ	8.46	2.194 (0.21)
Aug-cc-pVTZ	1.77	2.170 (0.21)
cc-pVQZ	3.18	2.178 (0.21)

Table S6. pK_a values obtained for PO-I-H and PO-II-H through direct calculation (pK_a^1) and with the isodesmic method (pK_a^2). Data calculated by B3LYP and M06-2X functionals, with different basis sets for all atoms but Fe (LANL2DZ), and PCM at T = 298.15 K. The most favourable spin multiplicities were used; PO-I and PO-I-H with S = 1/2, and PO-II and PO-II-H with S = 1.

**B3LYP**	**PO-I-H**		**PO-II-H**	
	**pK_a^1**	**pK_a^2**	**pK_a^1**	**pK_a^2**
6-31G(d,p)	–1.9	–18.2	11.2	–5.1
6-311G(2d,2p)	–1.4	–14.1	8.8	–3.9
6-311++G(2d,2p)	–1.9	–9.0	8.2	1.0
cc-pVDZ	–4.2	–17.7	8.5	–5.0
cc-pVTZ	–1.3	–11.6	8.0	–2.3
Aug-cc-pVTZ	–1.5	–9.0	7.6	0.0
cc-pVQZ	–1.4	–10.1	7.7	–1.1
**M06-2X**	**PO-I-H**		**PO-II-H**	
	**pK_a^1**	**pK_a^2**	**pK_a^1**	**pK_a^2**
6-31G(d,p)	18.2	3.2	23.3	8.3
6-311G(2d,2p)	9.7	–2.3	22.4	10.5
6-311++G(2d,2p)	9.3	2.0	22.4	15.1
cc-pVDZ	16.2	3.4	21.0	8.2
cc-pVTZ	7.9	–1.4	21.3	12.0
Aug-cc-pVTZ	8.2	1.1	21.3	14.2
cc-pVQZ	8.7	0.5	21.4	13.2

Table S7. Wavelength (nm) and molar absorption coefficient ($cm^{-1} \cdot mol^{-1} \cdot dm^3$) of the Soret band obtained for all considered species and calculated with the B3LYP/cc-pVDZ computational method.

Species	Spin	λ	ε
Fe(III)-PO	5/2	368	69925
Fe(III)-PO-H_2O	1/2	372	71844
Fe(III)-PO-H	1/2	369	19822
Fe(II)-PO	2	387	61976
Fe(II)-PO-H_2O	2	388	59915
Fe(II)-PO-H	1	373	37872
PO-I	1/2	373	57952
PO-I-H_2O	1/2	375	51619
PO-I-H	1/2	400	3433
PO-II	1	383	63192
PO-II-H_2O	1	384	59793
PO-II-H	1	370	56965

Figure S1. Calculated excitation lines and the corresponding convoluted UV-vis absorption spectra at the Soret region obtained with the TD-DFT functional with the B3LYP/cc-pVDZ computational level at the most stable spin state for (A) non-protonated Fe(III)-PO (—), Fe(II)-PO (—), PO-I (—), and PO-II (—); (B) corresponding aquo complexes; and (C) protonated species.

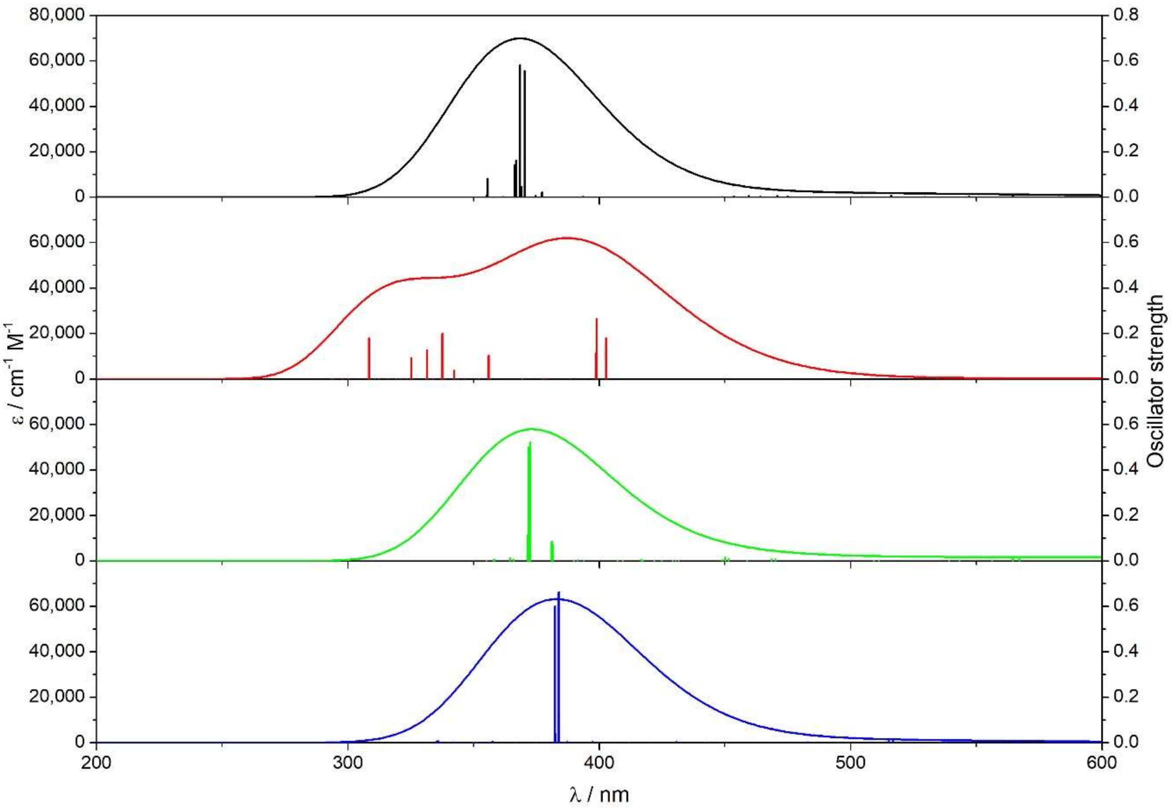

B

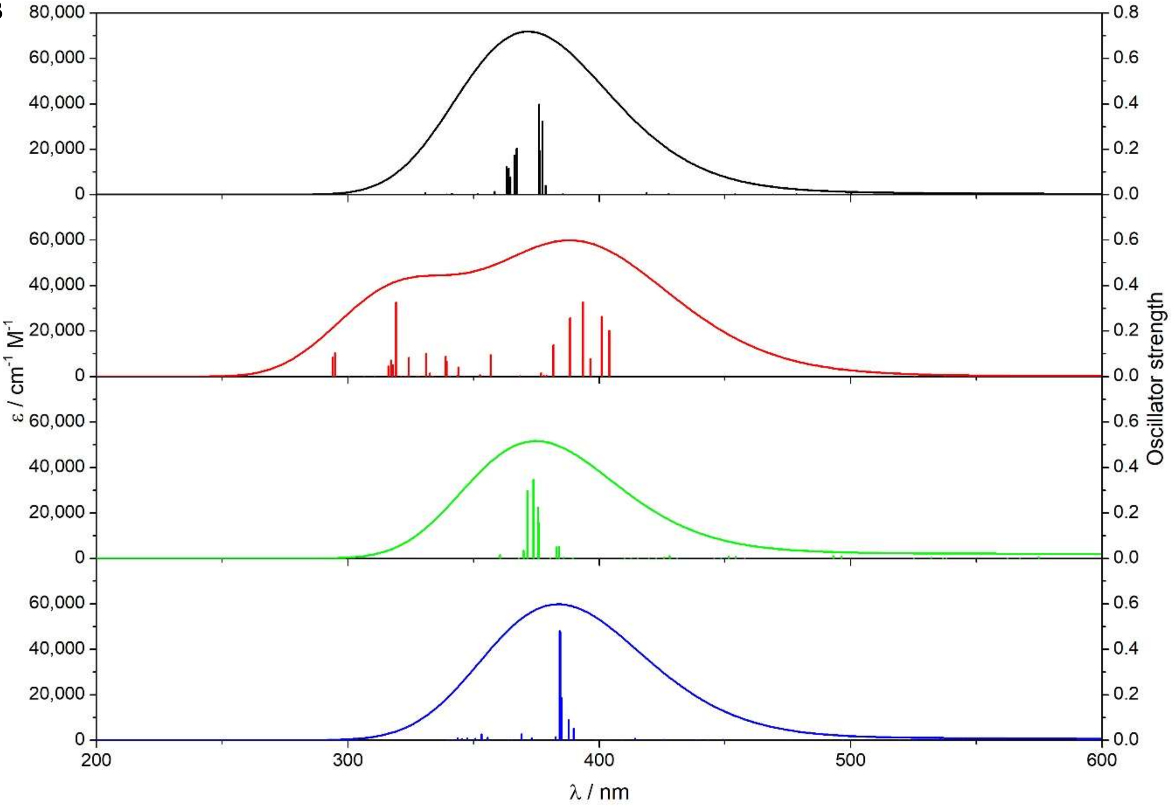

ε / cm⁻¹ M⁻¹
Oscillator strength
λ / nm

C

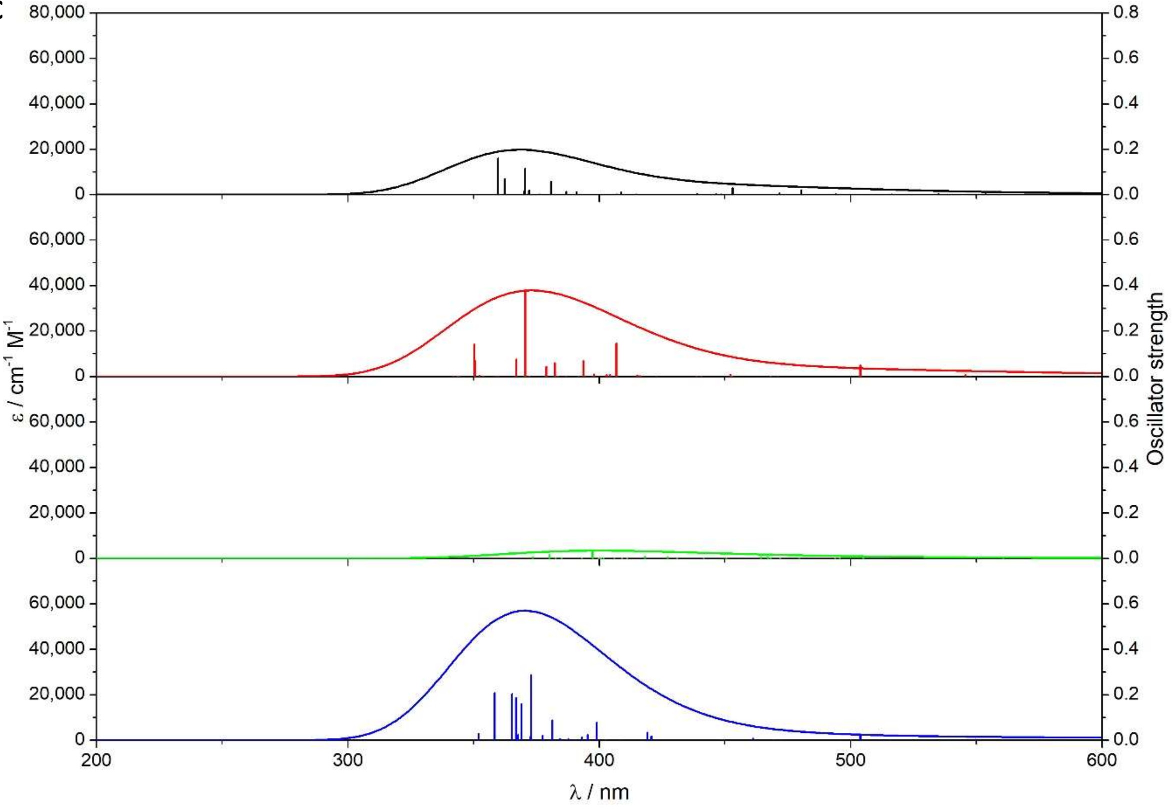

ε / cm⁻¹ M⁻¹
Oscillator strength
λ / nm

Table S8. Standard reduction potential ($E°$) values in mV relative to SHE calculated with direct and isodesmic methods, by B3LYP and M06-2X functionals, with different basis sets for all atoms but Fe (LANL2DZ), and PCM at T = 298.15 K. The most favourable spin multiplicities were used (as specified in parentheses).

	**Redox couple**					
	**Fe(III)-PO/Fe(II)-PO (5/2 – 2)**	**PO-I/Fe(III)-PO (1/2 – 5/2)**	**PO-I/PO-II (1/2 – 1)**	**PO-I/PO-II-H (1/2 – 1)**	**PO-II/Fe(III)-PO (1 – 5/2)**	**PO-II-H/Fe(III)-PO (1 – 5/2)**
**Direct method**						
**B3LYP**						
6-31G(d,p)	227	1136	592	1254	1679	1017
6-311G(2d,2p)	308	1279	825	1346	1733	1213
6-311++G(2d,2p)	365	1400	859	1345	1941	1456
cc-pVDZ	377	1066	741	1245	1391	886
cc-pVTZ	300	1324	859	1332	1790	1316
Aug-cc-pVTZ	121	1420	880	1328	1960	1512
cc-pVQZ	188	1388	876	1329	1900	1448
**M06-2X**						
6-31G(d,p)	652	2449	1403	2783	3496	2116
6-311G(2d,2p)	693	2328	1092	2419	3564	2236
6-311++G(2d,2p)	714	2452	1167	2490	3737	2414
cc-pVDZ	782	2405	1518	2761	3293	2050
cc-pVTZ	662	2358	1120	2380	3596	2336
Aug-cc-pVTZ	409	2468	1199	2461	3736	2475
cc-pVQZ	484	2443	1164	2431	3723	2456
**Isodesmic method**						
**B3LYP**						
6-31G(d,p)	17	858	382	1045	1470	807
6-311G(2d,2p)	–46	911	472	992	1380	860
6-311++G(2d,2p)	–110	1033	385	870	1467	982
cc-pVDZ	237	798	601	1105	1251	747
cc-pVTZ	–98	969	460	934	1392	918
Aug-cc-pVTZ	–373	1069	386	834	1466	1018
cc-pVQZ	–275	1036	414	867	1438	985
**M06-2X**						
6-31G(d,p)	–872	644	–121	1259	1973	593
6-311G(2d,2p)	–708	885	–310	1018	2162	834
6-311++G(2d,2p)	–812	939	–359	964	2211	888
cc-pVDZ	–698	621	39	1282	1813	570
cc-pVTZ	–770	955	–312	948	2164	904
Aug-cc-pVTZ	–1133	984	–342	919	2194	933
cc-pVQZ	–1033	989	–354	914	2206	938
